# *rac*-2-Amino-1,2-diphenyl­ethanol

**DOI:** 10.1107/S1600536812002000

**Published:** 2012-01-21

**Authors:** Ahmed Bari, Abdulrahman M. Al-Obaid, Seik Weng Ng

**Affiliations:** a Department of Pharmaceutical Chemistry, College of Pharmacy, King Saud University, Riyadh 11451, Saudi Arabia; b Department of Chemistry, University of Malaya, 50603 Kuala Lumpur, Malaysia; c Chemistry Department, Faculty of Science, King Abdulaziz University, PO Box 80203 Jeddah, Saudi Arabia

## Abstract

In the title compound, C_14_H_15_NO, the torsion angle about the two C*sp* ^3^ atoms adopts a partially eclipsed conformation [−61.5 (1)°]. The dihedral angle between the two rings is 48.1 (1)°. In the crystal, the mol­ecules are connected by O—H⋯N and N—H⋯O hydrogen bonds into zigzag chains running along [010]. One of the amino H atoms is not involved in hydrogen bonding.

## Related literature

For the use of chiral 2-amino-1,2-diphenyl­ethan-1-ol in organic synthesis, see: Masters & Hegedus (1993 ▶); Masters *et al.* (1991 ▶).
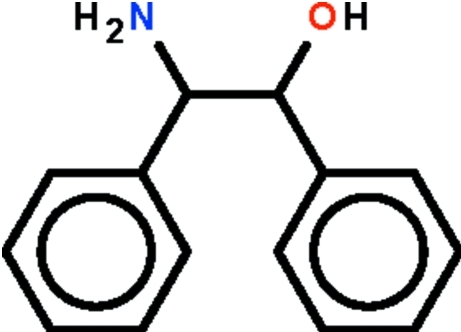


## Experimental

### 

#### Crystal data

C_14_H_15_NO
*M* *_r_* = 213.27
Monoclinic, 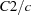

*a* = 26.6096 (6) Å
*b* = 5.3869 (1) Å
*c* = 17.1043 (4) Å
β = 114.689 (3)°
*V* = 2227.66 (8) Å^3^
*Z* = 8
Cu *K*α radiation
μ = 0.63 mm^−1^
*T* = 100 K
0.25 × 0.20 × 0.15 mm

#### Data collection

Agilent SuperNova Dual diffractometer with an Atlas detector
Absorption correction: multi-scan (*CrysAlis PRO*; Agilent, 2011 ▶) *T*_min_ = 0.859, *T*_max_ = 0.912
7706 measured reflections
2252 independent reflections
2139 reflections with *I* > 2σ(*I*)
*R*_int_ = 0.015

#### Refinement

*R*[*F* ^2^ > 2σ(*F* ^2^)] = 0.032
*wR*(*F* ^2^) = 0.085
*S* = 1.00
2252 reflections
158 parameters
H atoms treated by a mixture of independent and constrained refinement
Δρ_max_ = 0.30 e Å^−3^
Δρ_min_ = −0.21 e Å^−3^

### 

Data collection: *CrysAlis PRO* (Agilent, 2011 ▶); cell refinement: *CrysAlis PRO*; data reduction: *CrysAlis PRO*; program(s) used to solve structure: *SHELXS97* (Sheldrick, 2008 ▶); program(s) used to refine structure: *SHELXL97* (Sheldrick, 2008 ▶); molecular graphics: *X-SEED* (Barbour, 2001 ▶); software used to prepare material for publication: *publCIF* (Westrip, 2010 ▶).

## Supplementary Material

Crystal structure: contains datablock(s) global, I. DOI: 10.1107/S1600536812002000/bt5788sup1.cif

Structure factors: contains datablock(s) I. DOI: 10.1107/S1600536812002000/bt5788Isup2.hkl

Supplementary material file. DOI: 10.1107/S1600536812002000/bt5788Isup3.cml

Additional supplementary materials:  crystallographic information; 3D view; checkCIF report

## Figures and Tables

**Table 1:** Hydrogen-bond geometry (Å, °)

*D*—H⋯*A*	*D*—H	H⋯*A*	*D*⋯*A*	*D*—H⋯*A*
O1—H1⋯N1^i^	0.93 (2)	1.89 (2)	2.813 (1)	172 (2)
N1—H11⋯O1^ii^	0.91 (2)	2.38 (2)	3.178 (1)	148 (1)

Symmetry codes: (i) 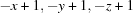; (ii) 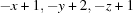.

**Fig. 1.**
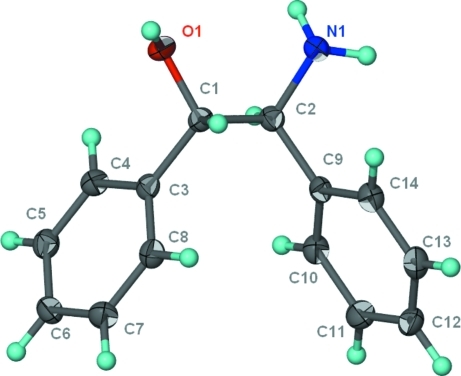
Anisotropic displacement ellipsoid plot (Barbour, 2001) of C14H15NO at the 70% probability level; hydrogen atoms are drawn as spheres of arbitrary radius.

## supplementary crystallographic information

### Comment

Optically active 2-amino-1,2-diphenylethanol is commonly used for palladium-assisted chiral tandem alkylation and carbonylative coupling reactions (Masters & Hegedus, 1993; Masters *et al.*, 1991). The crystal structure of either one of the chiral enantiomers has not been reported although the crystal structures of several 2-ammonium-1,2-diphenylethanol carboxylates have been reported. The crystal structure of the racemic 2-amino-1,2-diphenylethanol (Scheme I) is presented here.

The aromatic rings of the ethyl chain of staggered, the twist being 48.1 (1) ° (Fig. 1). The hydroxy group is hydrogen-bond donor to the amino group of an adjacent molecule; the amino group is hydrogen-bond donor to the hydroxy group of another molecule. The hydrogen bonds generate a linear chain running along [0 1 0] (Table 1). The amino group uses only one H atom to form a hydrogen bond.

### Experimental

The compound was obtained commercially, and crystals were grown from its solution in ethanol.

### Refinement

Carbon-bound H-atoms were placed in calculated positions [C–H 0.95 to 1.0 Å, *U*_iso_(H) 1.2 to 1.5*U*_eq_(C)] and were included in the refinement in the riding model approximation.

The amino and hydroxy H-atoms were located in a difference Fourier map, and were freely refined.

### Crystal data

**Table:**

C_14_H_15_NO	*F*(000) = 912
*M**_r_* = 213.27	*D*_x_ = 1.272 Mg m^−^^3^
Monoclinic, *C*2/*c*	Cu *K*α radiation, λ = 1.54184 Å
Hall symbol: -C 2yc	Cell parameters from 5244 reflections
*a* = 26.6096 (6) Å	θ = 2.8–74.2°
*b* = 5.3869 (1) Å	µ = 0.63 mm^−^^1^
*c* = 17.1043 (4) Å	*T* = 100 K
β = 114.689 (3)°	Prism, colorless
*V* = 2227.66 (8) Å^3^	0.25 × 0.20 × 0.15 mm
*Z* = 8	

### Data collection

**Table:**

Agilent SuperNova Dual diffractometer with an Atlas detector	2252 independent reflections
Radiation source: SuperNova (Cu) X-ray Source	2139 reflections with *I* > 2σ(*I*)
Mirror	*R*_int_ = 0.015
Detector resolution: 10.4041 pixels mm^-1^	θ_max_ = 74.4°, θ_min_ = 3.7°
ω scan	*h* = −27→32
Absorption correction: multi-scan (CrysAlis PRO; Agilent, 2011)	*k* = −5→6
*T*_min_ = 0.859, *T*_max_ = 0.912	*l* = −21→20
7706 measured reflections	

### Refinement

**Table:**

Refinement on *F*^2^	Secondary atom site location: difference Fourier map
Least-squares matrix: full	Hydrogen site location: inferred from neighbouring sites
*R*[*F*^2^ > 2σ(*F*^2^)] = 0.032	H atoms treated by a mixture of independent and constrained refinement
*wR*(*F*^2^) = 0.085	*w* = 1/[σ^2^(*F*_o_^2^) + (0.0452*P*)^2^ + 1.7088*P*] where *P* = (*F*_o_^2^ + 2*F*_c_^2^)/3
*S* = 1.00	(Δ/σ)_max_ = 0.001
2252 reflections	Δρ_max_ = 0.30 e Å^−^^3^
158 parameters	Δρ_min_ = −0.21 e Å^−^^3^
0 restraints	Extinction correction: *SHELXL97* (Sheldrick, 2008), Fc^*^=kFc[1+0.001xFc^2^λ^3^/sin(2θ)]^-1/4^
Primary atom site location: structure-invariant direct methods	Extinction coefficient: 0.0028 (2)

### Fractional atomic coordinates and isotropic or equivalent isotropic displacement parameters (Å^2^)

**Table:**

	*x*	*y*	*z*	*U*_iso_*/*U*_eq_	
O1	0.54033 (3)	0.71776 (14)	0.48409 (4)	0.01865 (19)	
N1	0.49273 (3)	0.74451 (17)	0.59788 (6)	0.0188 (2)	
C1	0.57362 (4)	0.64493 (18)	0.57066 (6)	0.0155 (2)	
H1A	0.5680	0.4635	0.5765	0.019*	
C2	0.55297 (4)	0.78952 (18)	0.62945 (6)	0.0158 (2)	
H2	0.5593	0.9708	0.6247	0.019*	
C3	0.63447 (4)	0.68973 (18)	0.59384 (6)	0.0153 (2)	
C4	0.65167 (4)	0.89815 (19)	0.56318 (6)	0.0178 (2)	
H4	0.6251	1.0145	0.5278	0.021*	
C5	0.70746 (4)	0.93689 (19)	0.58397 (6)	0.0192 (2)	
H5	0.7188	1.0799	0.5630	0.023*	
C6	0.74672 (4)	0.7676 (2)	0.63521 (6)	0.0195 (2)	
H6	0.7848	0.7934	0.6487	0.023*	
C7	0.73012 (4)	0.56042 (19)	0.66658 (6)	0.0205 (2)	
H7	0.7568	0.4444	0.7020	0.025*	
C8	0.67424 (4)	0.52294 (19)	0.64605 (6)	0.0179 (2)	
H8	0.6631	0.3815	0.6680	0.021*	
C9	0.58301 (4)	0.71185 (18)	0.72263 (6)	0.0155 (2)	
C10	0.62569 (4)	0.85732 (19)	0.77983 (6)	0.0187 (2)	
H10	0.6360	1.0050	0.7599	0.022*	
C11	0.65356 (4)	0.7899 (2)	0.86577 (6)	0.0222 (2)	
H11A	0.6827	0.8911	0.9040	0.027*	
C12	0.63889 (4)	0.5752 (2)	0.89591 (6)	0.0215 (2)	
H12A	0.6576	0.5300	0.9548	0.026*	
C13	0.59667 (4)	0.42707 (19)	0.83945 (7)	0.0208 (2)	
H13	0.5866	0.2794	0.8597	0.025*	
C14	0.56900 (4)	0.49391 (19)	0.75318 (6)	0.0187 (2)	
H14	0.5404	0.3905	0.7148	0.022*	
H1	0.5299 (6)	0.572 (3)	0.4526 (11)	0.044 (4)*	
H11	0.4750 (6)	0.857 (3)	0.5560 (9)	0.029 (3)*	
H12	0.4821 (6)	0.777 (3)	0.6421 (9)	0.029 (3)*	

### Atomic displacement parameters (Å^2^)

**Table:**

	*U*^11^	*U*^22^	*U*^33^	*U*^12^	*U*^13^	*U*^23^
O1	0.0175 (3)	0.0230 (4)	0.0130 (3)	−0.0004 (3)	0.0041 (3)	0.0008 (3)
N1	0.0144 (4)	0.0250 (5)	0.0170 (4)	0.0014 (3)	0.0066 (3)	0.0016 (3)
C1	0.0156 (4)	0.0167 (5)	0.0133 (4)	−0.0006 (4)	0.0052 (4)	0.0004 (3)
C2	0.0149 (5)	0.0162 (5)	0.0165 (5)	−0.0004 (3)	0.0067 (4)	0.0005 (3)
C3	0.0169 (5)	0.0171 (5)	0.0126 (4)	−0.0008 (4)	0.0069 (4)	−0.0029 (3)
C4	0.0183 (5)	0.0176 (5)	0.0166 (4)	0.0008 (4)	0.0064 (4)	0.0010 (4)
C5	0.0212 (5)	0.0192 (5)	0.0183 (5)	−0.0036 (4)	0.0094 (4)	−0.0003 (4)
C6	0.0156 (5)	0.0241 (5)	0.0196 (5)	−0.0015 (4)	0.0082 (4)	−0.0021 (4)
C7	0.0186 (5)	0.0221 (5)	0.0204 (5)	0.0039 (4)	0.0077 (4)	0.0028 (4)
C8	0.0202 (5)	0.0172 (5)	0.0179 (5)	0.0001 (4)	0.0097 (4)	0.0012 (4)
C9	0.0154 (4)	0.0175 (5)	0.0162 (5)	0.0027 (4)	0.0090 (4)	−0.0003 (4)
C10	0.0202 (5)	0.0189 (5)	0.0188 (5)	−0.0012 (4)	0.0099 (4)	−0.0007 (4)
C11	0.0208 (5)	0.0274 (5)	0.0176 (5)	−0.0015 (4)	0.0073 (4)	−0.0032 (4)
C12	0.0221 (5)	0.0288 (6)	0.0156 (5)	0.0067 (4)	0.0097 (4)	0.0031 (4)
C13	0.0239 (5)	0.0204 (5)	0.0231 (5)	0.0038 (4)	0.0148 (4)	0.0040 (4)
C14	0.0187 (5)	0.0188 (5)	0.0205 (5)	−0.0003 (4)	0.0101 (4)	−0.0007 (4)

### Geometric parameters (Å, °)

**Table:**

O1—C1	1.4266 (11)	C6—C7	1.3878 (14)
O1—H1	0.928 (18)	C6—H6	0.9500
N1—C2	1.4822 (12)	C7—C8	1.3926 (14)
N1—H11	0.907 (15)	C7—H7	0.9500
N1—H12	0.927 (15)	C8—H8	0.9500
C1—C3	1.5169 (13)	C9—C10	1.3903 (14)
C1—C2	1.5433 (13)	C9—C14	1.3974 (14)
C1—H1A	1.0000	C10—C11	1.3900 (14)
C2—C9	1.5133 (12)	C10—H10	0.9500
C2—H2	1.0000	C11—C12	1.3867 (15)
C3—C8	1.3910 (14)	C11—H11A	0.9500
C3—C4	1.3947 (14)	C12—C13	1.3877 (15)
C4—C5	1.3903 (14)	C12—H12A	0.9500
C4—H4	0.9500	C13—C14	1.3934 (14)
C5—C6	1.3881 (14)	C13—H13	0.9500
C5—H5	0.9500	C14—H14	0.9500
			
C1—O1—H1	105.9 (10)	C7—C6—H6	120.2
C2—N1—H11	107.7 (9)	C5—C6—H6	120.2
C2—N1—H12	108.9 (8)	C6—C7—C8	119.86 (9)
H11—N1—H12	106.3 (12)	C6—C7—H7	120.1
O1—C1—C3	111.09 (7)	C8—C7—H7	120.1
O1—C1—C2	107.56 (7)	C3—C8—C7	120.93 (9)
C3—C1—C2	112.40 (8)	C3—C8—H8	119.5
O1—C1—H1A	108.6	C7—C8—H8	119.5
C3—C1—H1A	108.6	C10—C9—C14	118.53 (9)
C2—C1—H1A	108.6	C10—C9—C2	120.14 (9)
N1—C2—C9	110.70 (8)	C14—C9—C2	121.33 (9)
N1—C2—C1	107.78 (7)	C11—C10—C9	121.00 (10)
C9—C2—C1	111.81 (8)	C11—C10—H10	119.5
N1—C2—H2	108.8	C9—C10—H10	119.5
C9—C2—H2	108.8	C10—C11—C12	120.15 (10)
C1—C2—H2	108.8	C10—C11—H11A	119.9
C8—C3—C4	118.71 (9)	C12—C11—H11A	119.9
C8—C3—C1	120.51 (9)	C13—C12—C11	119.52 (9)
C4—C3—C1	120.78 (9)	C13—C12—H12A	120.2
C5—C4—C3	120.50 (9)	C11—C12—H12A	120.2
C5—C4—H4	119.7	C12—C13—C14	120.29 (10)
C3—C4—H4	119.7	C12—C13—H13	119.9
C4—C5—C6	120.30 (9)	C14—C13—H13	119.9
C4—C5—H5	119.8	C13—C14—C9	120.50 (9)
C6—C5—H5	119.8	C13—C14—H14	119.7
C7—C6—C5	119.68 (9)	C9—C14—H14	119.7
			
O1—C1—C2—N1	54.01 (9)	C1—C3—C8—C7	178.98 (9)
C3—C1—C2—N1	176.61 (8)	C6—C7—C8—C3	0.44 (15)
O1—C1—C2—C9	175.90 (7)	N1—C2—C9—C10	−139.20 (9)
C3—C1—C2—C9	−61.50 (10)	C1—C2—C9—C10	100.61 (10)
O1—C1—C3—C8	−142.45 (9)	N1—C2—C9—C14	41.13 (12)
C2—C1—C3—C8	96.96 (10)	C1—C2—C9—C14	−79.06 (11)
O1—C1—C3—C4	37.40 (12)	C14—C9—C10—C11	−0.70 (14)
C2—C1—C3—C4	−83.18 (10)	C2—C9—C10—C11	179.61 (9)
C8—C3—C4—C5	0.49 (14)	C9—C10—C11—C12	−0.23 (15)
C1—C3—C4—C5	−179.37 (9)	C10—C11—C12—C13	0.73 (15)
C3—C4—C5—C6	0.34 (15)	C11—C12—C13—C14	−0.30 (15)
C4—C5—C6—C7	−0.79 (15)	C12—C13—C14—C9	−0.65 (15)
C5—C6—C7—C8	0.40 (15)	C10—C9—C14—C13	1.14 (14)
C4—C3—C8—C7	−0.88 (14)	C2—C9—C14—C13	−179.18 (9)

### Hydrogen-bond geometry (Å, °)

**Table:**

*D*—H···*A*	*D*—H	H···*A*	*D*···*A*	*D*—H···*A*
O1—H1···N1^i^	0.93 (2)	1.89 (2)	2.813 (1)	172 (2)
N1—H11···O1^ii^	0.91 (2)	2.38 (2)	3.178 (1)	148 (1)

Symmetry codes: (i) −*x*+1, −*y*+1, −*z*+1; (ii) −*x*+1, −*y*+2, −*z*+1.
